# The use of Simulated Observations in Medical Simulation and its effect on perceived realism: A pilot project

**DOI:** 10.12688/mep.19719.1

**Published:** 2023-09-01

**Authors:** James Ainsworth, Sounder Perumal, Suresh Pillai

**Affiliations:** 1 Morriston Hospital, Swansea, SA6 6NL, UK

**Keywords:** Simulation, medical simulation, medical education, simulation fidelity, perceived realism

## Abstract

**Introduction**

Simulation is an effective teaching method with increasing growth and recognition and refers to the artificial representation of a real-life scenario. The aim of this study was to compare simulation with and without the use of a simulated observations monitor and to investigate differences in students’ impression of realism, engagement, learning, and enjoyment.

**Methods**

Simulation sessions were delivered to second and third-year Swansea University Medical Students, and a total of 15 students were included. Students carried out 2-3 scenarios each with and without the use of a simulated observations monitor. Data collection was conducted via student surveys and a joint interview.

**Results**

All students had an increased sense of realism with the use of the simulated observations monitor, feeling a closer resemblance to what would be experienced in clinical practice. They felt this improved their learning, making them more prepared for the real-life scenario. The monitor was more dynamic, responding to their interventions, helping them maintain focus and engagement throughout. A key theme was the reduction of interruptions or deviations from the scenario to communicate with the examiner or ask for observations. The visual and audible affects provided additional stimuli, adding to the realistic nature of the simulation.

**Discussion**

Simulation has been shown to be a useful education tool, but there is less evidence to support the use of higher fidelity over lower fidelity simulation. The terms are often used inconsistently, and many factors affect the students’ perceived sense of realism. This study shows that the addition of a simple device such as the simulated observations monitor can produce a higher level of fidelity, particularly in terms of the stimuli provided and student perceptions of realism, which may be effective in improving engagement with the simulation, learning, and aid recall when presented with similar scenarios in a real-life situation.

## Introduction

Simulation teaching is an educational tool used within medical school and specialty training, with increasing growth and recognition of the potential benefits in medical education. It is a method that is not unique to medicine, and is a technique used in other professions for many years, such as the aviation and aerospace industry, and in the military ( Al-Elq, 2010). According to Al-Elq 2010, “Simulation is a generic term that refers to an artificial representation of a real world process to achieve educational goals through experiential learning”. In medical education, simulation is a method of teaching where a clinical scenario is replicated in a safe and controlled environment, with the aim of promoting the acquisition of skills and knowledge through stimulating the expected behaviours. It provides opportunities for students to practice skills without the fear of making mistakes or causing harm to a real patient. Any mistakes made during a simulation session provide opportunities for feedback and development ( Al-Elq, 2010). It is used in both undergraduate and postgraduate medical training, and is an important tool in specialty training such as anaesthetics, where it is useful in the development of clinical and communication skills, particularly during emergency situations. Simulation also allows the facilitator to create scenarios rarely encountered in practice e.g. malignant hyperthermia ( Maran & Glavin, 2003; So *et al.*, 2019), and is an effective tool for inter-professional education and collaboration ( So *et al.*, 2019).

There is increasing emphasis in modern medical curricula not only on the acquisition of knowledge, but on its application in practice, with competence in clinical skills being essential in a newly qualified doctor. This includes physical examination, history taking and diagnostic skills, resuscitation, and procedural skills, but also core skills such as communication skills, problem solving, clinical reasoning, teamwork and leadership. Organisational skills, time management, and prioritisation of tasks are also important. How best to help develop all of these skills, in addition to the vast amount of knowledge required to qualify as a doctor, is the challenge faced by medical school curricula ( Al-Elq, 2010). Incorporating simulation teaching is shown to be associated with improved outcomes in terms of knowledge, skills and behaviours ( Issenberg *et al.*, 2005; Norman *et al.*, 2012; So *et al.*, 2019; Tun *et al.*, 2015). Students are more frequently thought of as adult learners, and simulation provides the learners with more control over their learning experience ( Norman *et al.*, 2012; So *et al.*, 2019), as well as incorporating the concept of “situated cognition” ( Norman *et al.*, 2012) which suggests that better learning is achieved the closer the learning context to the context where it will be applied.

There is a growing interest in high fidelity simulation, but there is less evidence that higher fidelity simulation is more beneficial in terms of performance outcomes when compared with lower fidelity simulation ( Kardong-Edgren *et al.*, 2007; Massoth *et al.*, 2019; Norman *et al.*, 2012). Simulation may differ in how ‘realistic’ it is, also called the ‘fidelity’ of simulation ( Al-Elq, 2010). The term may however cause confusion on what exactly defines ‘high’ or ‘low’ fidelity and is inconsistently used, but is generally thought to describe the extent to which the simulation resembles the real life scenario and tasks that are being simulated ( Maran & Glavin, 2003; Tun *et al.*, 2015). Both low and high fidelity simulation may have a role in medical education. Low fidelity simulators provide the basis of many medical examinations, such as OSCEs ( Maran & Glavin, 2003). Lower fidelity simulation, lacking situational context may be useful for focussed learning of simple tasks, for example procedural skills (e.g. cannulation) ( Al-Elq, 2010). Higher fidelity simulation has the aim of being as realistic as possible, and may be more complex in nature. This may be highly variable, but generally is seen as having a real life scenario or context, and a whole body manikin, with some simulation centres having a huge amount of technology allowing manikins to closer resemble living patients, with the ability to communicate and interact with the mannequin, display physiological signs, and withstand interventions ( Al-Elq, 2010; Massoth *et al.*, 2019). However, does this additional technology actually increase the learner’s sense of realism when carrying out the simulation? And how does this affect learning and outcomes? A greater understanding of this is important, given the additional demands of running such high fidelity simulation (cost, equipment, space, time, staff training and numbers, etc.), and is this additional cost and requirements necessary to produce effective results? Further research is needed in this area, given the current controversial nature of the benefits of high fidelity simulators ( Al-Elq, 2010; Massoth *et al.*, 2019; So *et al.*, 2019).

It is important to note that the ‘fidelity’ of the simulation and the students’ sense of realism is not only influenced by how ‘realistic’ the environment is and its resemblance to the real world, but also how realistic the scenario is felt to be, the expected actions of the learner, and how the scenario unfolds during the course of the simulation. Flexibility from the facilitator can play an important role here. These factors must all be taken into account when planning and delivering a simulation teaching session. Pre-briefing the artificial nature of the simulation session or “fiction contract” ( So *et al.*, 2019) and the expectation to commit to the simulation as if it were a real clinical scenario may actually contribute to their perceived level of realism then during the activity. The concept of ‘engineering fidelity’, the extent to which the environment and task replicates the real life scenario, and ‘psychological fidelity’, i.e. is the simulation able to promote the learner to carry out specific behaviours required, which may not essentially be dependent on how ‘realistic’ the simulation appears ( Maran & Glavin, 2003; Norman *et al.*, 2012). Simple simulation or lower fidelity may still provide a high level of psychological fidelity ( Norman *et al.*, 2012). One interpretation of fidelity is that a simulation may therefore be seen as being high fidelity if the right cues and stimuli are provided, prompting the desired behaviour ( Tun *et al.*, 2015).

This study provides a comparison between simulation teaching with and without the use of a simulated observations monitor (SOM), and a simple comparison of a lower and higher fidelity simulation. The aim of the SOM here is to negate the need for continuous interruptions by the examiner or facilitator to deliver the observations, but the visual and audible effects may add significantly to the student’s sense of realism. This idea of perceived realism and the student’s level of it with the addition of small changes is discussed in this study.

## Methods

This was a prospective pilot study that enrolled 15 second and third year Swansea University Medical School post graduate entry medical students. Only students in their second or third year of study from Swansea University Medical School graduate entry medicine programme were included in the study. There were no other specific inclusion/exclusion criteria beyond this. Students were included on a first come first served basis, with those that responded first to participant recruitment emails being included in the study. The Medical Research Council (MRC) tool was used in order to determine if NHS REC review was required for this pilot study, which indicated that REC review was not required for sites in Wales. Simulation scenarios were written with an acutely unwell patient, at an appropriate level for second and third year graduate entry medical students. Scenarios included medical emergencies, such as: acute myocardial infarction (MI); seizure; anaphylaxis; acute asthma or chronic obstructive pulmonary disease (COPD) exacerbation; diabetic ketoacidosis (DKA); sepsis; etc., and surgical emergencies, such as: bowel perforation; trauma; wound infection. The student was given written information with a brief patient background and instructions, then asked to enter the room and start the scenario immediately. Each scenario ran for 10 – 15 minutes. Information for the facilitator included observations at the start of the scenario, and changes as the scenario developed, which could vary depending on interventions carried out by the student.

Additional equipment and props consisted of: a sim mannequin (head and torso but with no electronic features); basic ward equipment such as cannulas, syringes, blood bottles, a choice of fluids; and drug charts which the students could prescribe any medication given.

Basic learning aims were created: the main one being to carry out an ABCDE assessment of an acutely unwell patient, and to practice their structured approach to the assessment and management; problem solving and working under pressure were additional aims. Group discussion or debrief was done at the end of the session, allowing for feedback and for the students to analyse their own performance.

Multiple sessions were organised and facilitated by the author. Sessions were delivered in small groups with 2 – 4 students attending per session.

The aim of the study was to compare simulation with and without the use of a simulated observations monitor, and to investigate differences in the student’s impression of realism, engagement with the simulation, learning, and enjoyment or preference.

Students carried out 2 – 3 scenarios each without the use of the simulated observations monitor, with all observations at the start and any changes during the scenario being delivered verbally by the facilitator. They would then carry out similar simulation scenarios with the use of the simulated observations monitor, which could be seen clearly on a screen, this also had the benefit of providing visual and audible cues, as with a real patient bedside observations monitor.

The SimMon app ( Castle, 2018) was used to display the ‘patients’ observations during the scenario. An Apple iPad was used as the display monitor, which was able to display: respiratory rate (RR); oxygen saturations (SpO2); heart rate (HR) including an electrocardiogram (ECG) trace; blood pressure (BP); and end tidal carbon dioxide (ETCO2). Different waveforms for the vital signs were also possible to display, such as a damp SpO2 trace, and different ECG rhythms, such as an ST elevation MI (STEMI), atrial fibrillation (AF), or a cardiac arrest rhythm such as ventricular tachycardia (VT) or ventricular fibrillation (VF). The Apple iPad was linked to another device allowing the facilitator to alter the readings effortlessly during the course of the scenario, and respond to interventions carried out by the students or make changes as the scenario progressed, without the need for further communication with the student.

Data was gathered by two methods:

- Joint interview with 2 students
- A 9 question online survey with 10 respondents

A link to the online survey was sent to the students following completion of both simulations. This was done using an anonymous online Survey Monkey. Once all students had responded to the survey the results were reviewed. The joint interview was facilitated by the researcher (Dr James Ainsworth) immediately following completion of the simulation, with two students. The interview was semi-structured, using a pre-formed set of questions, which were similar to those used in the online survey, to allow collection of additional qualitative data and a more in depth analysis. The aim was to explore and compare students perceptions or feelings in both simulations (with and without the simulated observations). Audio from the interview was recorded, and was manually transcribed by the author, ensuring all identifiable data was removed.

All simulation and the interview were carried out in the medical education centre within Morriston Hospital. Only the researcher (Dr James Ainsworth) and the students attending the simulation were present at the time.

The survey and interview questions attempted to gather information on the following themes, around which the data were also organised: Sense of realism; Engagement with the simulation; Learning; Enjoyment/Feelings, which allow easier interpretation of the results.

All students attending teaching sessions agreed for any data collected to be used for educational research, with signed informed consent. Any data collected from the surveys or focussed group was anonymised, with no names or other details included. The survey responses were also all anonymised, and included a question confirming that the respondent was happy for the answers given to be used for research. The results from this study will be used to design a larger study.

## Results

### Sense of realism

**Figure 1.**
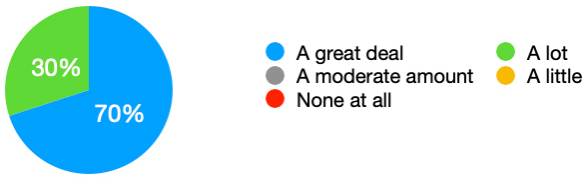
Sense of realism: Pie chart showing the extent to which the simulated observations monitor increased students perceived realism. All students felt their sense of realism was increased, with 70% stating their sense of realism was increased a great deal.

All respondents to the survey felt that the use of the simulated observation increased their sense of realism whilst in the simulation scenario. Figure 1 demonstrated the results clearly, and that 70% of students felt that their sense of realism was increased a great deal. All students felt that their sense of realism was either ‘A lot’ or ‘A great deal’ more with the simulated observations monitor.

Written responses by the students in the survey demonstrated an increased sense of realism. “Much more realistic in real time, to see the results after action” [Respondent 3]. “Real time updates, allows you to react instantly and see changes from your interventions” [Respondent 6]. “It was much more realistic as you could look at the observations in live time and react to them as they changed rather than asking for an update from the person running the sim” [Respondent 8]. There was clearly a feeling that this was more realistic, with a closer resemblance to what would be experienced in clinical practice. “Getting used to looking at the observations and interpreting them, provides better clinical picture than just hearing the values which can go in one ear and out the other” [Respondent 7]. There was a feeling of things happening in real time, and allowing them to respond to changes more quickly, and to more easily assess the response to their interventions.

The feedback in the interview was similar, with both students stating their sense of realism was increased. Interviewee 1 commented on the audible sounds of the observations making the scenario feel more realistic. Interviewee 1 again commented on the monitor making it easier to adapt to changes in real time.

### Engagement with the simulation

**Figure 2.**
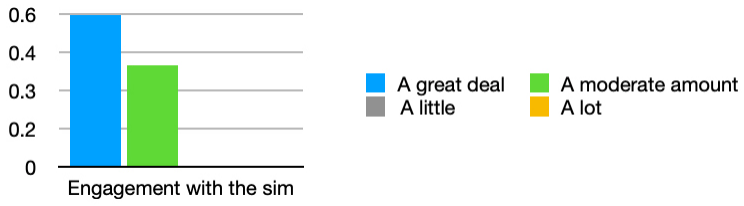
Engagement with the simulation: Bar graph showing the effect of the simulated observations monitor on students engagement with the simulation monitor. All students either stated that their engagement was increased a moderate amount, or a great deal.

All students felt their engagement with the simulation was increased with the simulated observations monitor (see Figure 2). 60% of students in the survey stated that their engagement improved a great deal, “With monitor it was much easier to stay engaged” [Respondent 9].

This is clearly partly due to the increased sense of realism felt by the students during the simulation scenario, “Made it feel more real, and that it wasn't just a dummy lying in bed” [Respondent 5], “Felt more ‘clinical’ and in control of the situation” [Respondent 7]. Students also stated that they felt they were better able to assess the results of their interventions, and to more easily monitor the changes (improvement or deterioration) in the patient’s status as the scenario progressed or following interventions.

Another key factor appears to be the flow through the scenario, and the reduced interruptions from the scenario to gain information from the facilitator regarding the status of the patient, “It didn’t break up the flow of the simulation by asking for observations, made it more like a real life situation” [Respondent 8]. Not having to ask the facilitator for information repeatedly throughout and whether any changes had occurred, particularly following the administration of a treatment or an intervention allowed the students to remain focussed and engaged throughout.

The feedback from the interview was positive, with similar information gathered as from the survey, “...we also disconnected less to talk to the examiner...” [Interviewee 2]. The interviewees commented on reacting to changes in the observations quicker, and being able to see or hear changes as they went along. “... was much easier to adapt in real time vs continually asking the person in charge of the sim has the heart rate changed, has the resp rate changed or what not” [Interviewee 1].

### Learning

**Figure 3.**
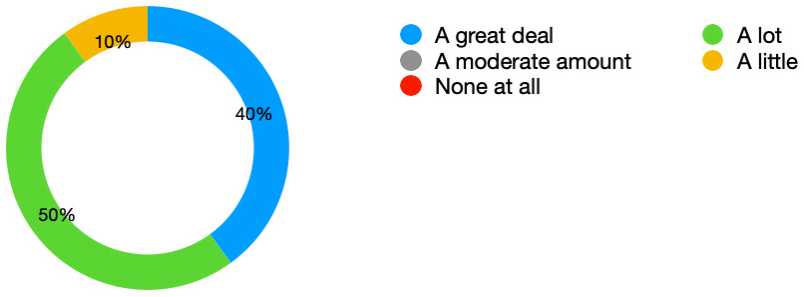
Learning: Pie chart displaying the extent to which students felt their learning improved as a result of having the simulated observations monitor. 0 students marked none at all or a moderate amount. 50% stated a lot and 40% stated a great deal.

The majority of students felt like their learning was improved by the use of the simulated observations monitor. Figure 3 displays the percentages as a chart. 0 students marked none at all or a moderate amount.

Students felt that the more realistic nature of the simulation session with the use of the simulated observation monitor improved their learning, by making them more prepared for a real life scenario, “Feels more realistic so builds confidence in clinical skill and looking at monitors on the wards - knowing how it fits together” [Respondent 7], “prepare for real life scenario” [Respondent 6]. “It gave me a more realistic example of how situations unfold and puts you under pressure to apply your knowledge. It also highlights areas you are less confident in very evidently” [Respondent 2].

One student commented that it helped solidify their ABCDE approach to the acutely unwell patient. Some commented on an improvement in focus through the scenario. “I felt they were conducive to my learning, I felt that the simulations really emphasised the importance of ABCDE assessments” [Respondent 5].

One student however felt that it made little difference to the outcome or their learning, but that it did make the scenario feel more realistic, and “...made the abcde progression smoother and made the sim less disjointed” [Respondent 8].

Similar data was gathered from the focus group, with Interviewee 1 stating that the more realistic the simulation training the more prepared you feel when faced with similar scenarios in real life. Interviewee 1 stated that they “... felt more focussed with it. Rather than just thinking this is a simulation session, because it was more realistic you were more focussed on the actual scenario presented in front of you rather just this is a teaching session or an examination session”.

### Enjoyment/Feelings

**Figure 4.**
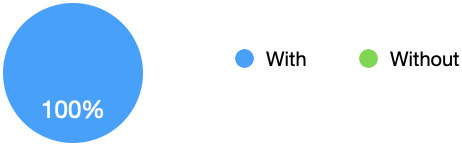
Enjoyment/Feelings: Pie chart showing that all students preferred the simulation with the use of the simulated observations monitor. All students (100%) stated that they preferred the simulation with the use of the simulated observations monitor.

All students in the survey (100%) stated that they preferred the simulation scenarios with the simulated observations monitor, again this was often due to the increased sense of realism during the simulation (see Figure 4). Being able to see the observations during the scenario continuously also meant that the students did not need to remember all the information delivered verbally by the facilitator, with one commenting that can actually add to the confusion during the scenario. “I quite enjoyed having it there just so I could keep looking at it and referring to it myself” [Interviewee 2]. Some did comment however on an increased feeling of pressure or anxiety, particularly when starting the simulation. Figure 2 shows that all students preferred the simulation with the use of the simulated observations monitor.

## Discussion

We can see from the literature that simulation is proven to be a useful educational technique in medical education, and is shown to be beneficial in developing clinical competence. Despite the growing popularity and ingenuity of simulation within medical education, the benefits and differences in outcomes of higher fidelity simulation in terms of training and development and patient safety is less clear. What exactly defines the fidelity of a simulation session or scenario is also variable between sources, with many factors requiring consideration that may contribute to the student’s sense of realism and commitment or engagement with the simulation.

This study shows that a simple measure such as the addition of a simulated observations monitor may significantly increase the learner’s perceived sense of realism. This may be a relatively simple intervention, but is sufficient to create a noticeable change in the fidelity of the simulation scenario. The presence of the monitor gave the students a feeling of closer resemblance to attending to a patient on the ward. All participants in this study (both the survey and the interview) stated that the addition of the simulated observation monitor increased their sense of realism. ‘Made it feel more real, and that it wasn't just a dummy lying in bed’ [Respondent 5]. The following quote by Respondent 9 is effective in conveying the increased feelings of realism: ‘More immersive, real time monitoring, not having to "check out of the scenario" for information from examiner’.

This may be largely due to the benefit of reduced interruptions during the scenario. The simulated observations monitor reduces the need for deviations from the scenario by negating the need for repeated communication with the facilitator, therefore helping maintain the student’s sense of realism throughout the session, “I think we also disconnected less to talk to the examiner. So it’s not ‘in out’ it’s just in there, there are the obs, and you don’t have to turn to the examiner and say what’s happening now what’s happening now” [Interviewee 2].

In addition, the visual effect of actually seeing the patients vital signs displayed on the monitor in real time, combined with auditory stimulation kin to that heard on the wards therefore being more similar to a clinical situation in which they might encounter in practice. Some students did comment on the benefits of being able to see the observations on the monitor continuously, and also in being able to hear the sounds. Students often appeared very receptive to audible changes in the monitor, created by changes in the vitals, such as an increase in heart rate or a change in tone with a decline in oxygen saturations. “Even having the background noise makes it a bit more realistic” [Interviewee 1]. “I like the fact that I could see the changes live during the simulation, felt more realistic - albeit the sounds it made was more heart-racing!” [Respondent 5].

Engagement with the simulation was higher during the sessions with the simulated observations monitor. “With monitor it was much easier to stay engaged” [Respondent 9]. The information given via the monitor is dynamic, allowing the facilitator to make changes to the patients’ vitals throughout the scenario, and respond to any interventions. This was appreciated by the students. “Much more realistic in real time, to see the results after action” [Respondent 3]. This allowed the students to maintain focus on the task at hand.

The students felt that the use of the simulated observations monitor also improved their learning. Removing the need to remember all the observations delivered at the start of the scenario and the mental effort required for non-essential tasks or learning, therefore decreasing the extrinsic cognitive load. It also allowed the students to refer back to the information during the scenario, and to monitor carefully for any changes. One student felt that having the monitor did not change what they learned, but made the simulation more realistic and the scenario ran more smoothly, less disjointed.

So which did the students prefer? Simulation is a useful learning tool, and creates a safe space to do practice emergency or rarely encountered scenarios. Simulation can however be a nerve-racking or stressful process for students, particularly when used as for examinations, or when being watched by colleagues. All students in this study stated that they preferred the simulation sessions using the SOM as opposed to similar sessions without it. It may also however add to the pressure felt whilst approaching or performing the sim, although one would hope this was due to that sense of realism and the feeling of a closer resemblance to being in a real life scenario or assessing an unwell patient. Students did comment on the additional pressure felt, but some felt that this added to their performance.

This study shows that the addition of a simple device such as the SOM can produce a higher level of fidelity, particularly in terms of the stimuli provided and student perceptions of realism, which may be effective in improving engagement or commitment with the simulation, learning, and aid medical student (and then junior doctor) recall then when presented with similar scenarios in a real life situation, or situated cognition ( Norman *et al.*, 2012). “with a simulation session, it’s an artificial environment, by having something that’s a bit more realistic it takes that artificialness away from it, so if you were presented with a scenario in real life you’re far more ready to deal with it as it was rather than trying to remember what you did in a sim session”. [Interviewee 1]. This feeling of helping the students ‘prepare for real life scenario’ [Respondent 6], may also increase motivation or intrinsic drive, with an increased feeling of relevance.

## Limitations

This was a pilot study with a small number of participants, and included medical students on the post-graduate entry course only from the second and third year, from one medical school. There was no follow up beyond this to assess for ongoing learning or proven changes in performance, with subjective data collected by student self-assessment and perceptions or feelings following the session.

## Data Availability

This project contains the following underlying data: -   Anonymised survey results.docx Figshare: Anonymised survey results.docx. https://doi.org/10.6084/m9.figshare.23599995.v1 ( Ainsworth, 2023a). -   Anonymised survey results (non-aggregated data) Figshare: Simulation Pilot Study anonymous survey data (non-aggregated). http://doi.org/10.6084/m9.figshare.23913021 ( Ainsworth, 2023b). -   Interview Transcript.docx Figshare: Interview Transcript.docx. https://doi.org/10.6084/m9.figshare.23599992.v1 ( Ainsworth, 2023c). Data are available under the terms of the Creative Commons Attribution 4.0 International license (CC-BY 4.0).
