# Supplementary material for: The use of Simulated Observations in Medical Simulation and its effect on perceived realism: A pilot project
Source: MedEdPublish (2016). 2024 May 30;13:66. Originally published 2023 Sep 1. [Version 2] doi: 10.12688/mep.19719.2 (PMC11153985; doi:10.12688/mep.19719.2)
Supplement: Peer review history

# Reviewer report: Reviewer response for version 2

**DOI:** 10.21956/mep.21841.r37307

**Published:** 2024-06-05

**Authors:** Anne D Souza

**Affiliations:** 1 Department of Anatomy, Kasturba Medical College, Manipal, Manipal Academy of Higher Education, Manipal, Karnataka, India

I have no further comments to make.

Have any limitations of the research been acknowledged?

Yes

Is the work clearly and accurately presented and does it cite the current literature?

Partly

If applicable, is the statistical analysis and its interpretation appropriate?

Yes

Are all the source data underlying the results available to ensure full reproducibility?

Yes

Is the study design appropriate and is the work technically sound?

Partly

Are the conclusions drawn adequately supported by the results?

Partly

Are sufficient details of methods and analysis provided to allow replication by others?

Yes

Reviewer Expertise:

Planning and designeing learning, Medical Ethics, 3D Printing in Anatomy Education, Simulation in medical education

I confirm that I have read this submission and believe that I have an appropriate level of expertise to confirm that it is of an acceptable scientific standard.

# Reviewer report: Reviewer response for version 1

**DOI:** 10.21956/mep.21125.r35561

**Published:** 2024-05-06

**Authors:** Stephanie Mansell

**Affiliations:** 1 University College London, London, England, UK

Thank you for the opportunity to review this manuscript. The authors should be congratulated on taking the time to share their work. This article describes how simulated monitoring devices impacts on the realism of simulation based education.

 I would encourage the authors to increase the clarity of their title, it should be made clear in the title the research is investigating the use of a simulated observation monitor.

 The authors should take the time to ensure their references are relevant and up to date. I would encourage them to read the work of Gaba. There are inconsistencies in the language used. The role of debrief is an essential aspect of simulation and should be included in the definition. Given the focus of the article on the difference between fidelity and realism the authors should be clear to define these terms. The definitions used currently are unclear.

 Of most concern is the lack of trial registration, I would have expected to see at least institutional registration of some description. Secondly it is concerning that there was no ethics sought. This is justified, however the authors refer to recruiting participants, consent and refers to the work as research. 

 The aims are repeated and are inconsistent, I would suggest the authors review the aims at the end of the introduction and those stated in the methods.

 Research has shown that the "sim effect" starts to be reduced and students are more able to suspend disbelief, with resulting increased fidelity after 3 scenarios. The authors state that participants completed 2-3 scenarios. The difference between 2 and 3 scenarios could impact on the results and I would encourage the authors to include this in their discussion.   

 The methods would have improved by randomizing the order in which participants were exposed to the simulated monitor. The impact of the order should be included in the discussion and this should be recognized as a limitation.

 There is a lack of detail on how the survey questions were designed. The survey questions are leading in nature and could have created confirmation bias, this should be recognized in the limitations and discussion. More detail should be provided in the methods. There should also be some justification on why validated surveys and outcome measures were not deployed. The response rate should be considered in the discussion.

 Additionally there is a lack of detail on the methods used for developing the interview questions or the methodologies used for data collection and analysis. I would encourage the authors to consider qualitative methodology. The provided transcript demonstrated that the interviews were very brief (3.5mins), which demonstrates a missed opportunity for data collection. The questions were leading in nature and more methodological rigor would have greatly improved this aspect of the research.

 The results of this study provide evidence at Kirkpatrick level 1, the authors should recognize this as a limitation and include this in the discussion. 

 Figure 4 is redundant and should be removed. Why is there different formatting between the figures, please ensure consistency in the formatting.

 New results are presented in the discussion, please ensure no new data is presented in the discussion. Please also ensure the quotes are relevant to the aims of the study.

 The discussion should cite the results of this study in the context of other literature. I would encourage the authors to source additional relevant references to increase the context of their results. The authors should avoid conjecture e.g. "students often appeared very receptive".

Have any limitations of the research been acknowledged?

Partly

Is the work clearly and accurately presented and does it cite the current literature?

Partly

If applicable, is the statistical analysis and its interpretation appropriate?

Yes

Are all the source data underlying the results available to ensure full reproducibility?

Partly

Is the study design appropriate and is the work technically sound?

Partly

Are the conclusions drawn adequately supported by the results?

No

Are sufficient details of methods and analysis provided to allow replication by others?

Partly

Reviewer Expertise:

Simulation-based education, respiratory physiotherapy, sleep and ventilation, airway clearance

I confirm that I have read this submission and believe that I have an appropriate level of expertise to state that I do not consider it to be of an acceptable scientific standard, for reasons outlined above.

# Reviewer report: Reviewer response for version 1

**DOI:** 10.21956/mep.21125.r35567

**Published:** 2024-01-25

**Authors:** Therese Gunn

**Affiliations:** 1 Queensland University of Technology, Brisbane, Queensland, Australia

Well done and I look forward to the extended version of this study beyond this pilot.

 You have indicated the importance of using authentic feedback devices (SOM) to enhance the learning of the medical students rather than a verbal list of observations.

 I feel there is a lack of background/definition of the SOM. At first I thought it referred to a method of educator observation of the student, but then realised it was the patient observation. I think this needs to be clearly defined/discussed in the introduction - perhaps use some of the description from the methods (7th paragraph) in the intro.

 The first paragraph in the introduction could benefit from providing some examples of the different simulation strategies (manikins, VR, role playing etc). Then when it comes to your study, define the simulation you are evaluating - manikin with real-time patient observation vs without.

 I would like to see some current literature in the introduction and evidencing your theories and aligning with your own conclusions. Especially your 2nd last paragraph in the discussion around the pressure of the task due to the realism.

 I have done a quick look at current literature (and I admit it was not thorough) but I include a few below that might offer some insights for your study.

Have any limitations of the research been acknowledged?

Yes

Is the work clearly and accurately presented and does it cite the current literature?

Partly

If applicable, is the statistical analysis and its interpretation appropriate?

Yes

Are all the source data underlying the results available to ensure full reproducibility?

Yes

Is the study design appropriate and is the work technically sound?

Partly

Are the conclusions drawn adequately supported by the results?

Partly

Are sufficient details of methods and analysis provided to allow replication by others?

Yes

Reviewer Expertise:

Simulation in education; healthcare education; learning design/pedagogy

I confirm that I have read this submission and believe that I have an appropriate level of expertise to confirm that it is of an acceptable scientific standard, however I have significant reservations, as outlined above.

## References

[1] : A randomised trial of the effectiveness of instructor versus automated manikin feedback for training junior doctors in life support skills. *Perspect Med Educ* .2021;10(2) : 10.1007/s40037-020-00631-y 95-100 10.1007/s40037-020-00631-y 33242153 PMC7952489

[2] : Use of simulation training to teach the ABCDE primary assessment: an observational study in a Dutch University Hospital with a 3-4 months follow-up. *BMJ Open* .2020;10(7) : 10.1136/bmjopen-2019-032023 e032023 10.1136/bmjopen-2019-032023 32636280 PMC7342483

[3] : Effects of high-fidelity simulation education on medical students' anxiety and confidence. *PLoS One* .2021;16(5) : 10.1371/journal.pone.0251078 e0251078 10.1371/journal.pone.0251078 33983983 PMC8118241

[4] : Exploring learners’ emotions and emotional profiles in simulation-based medical education. *Australasian Journal of Educational Technology* .2020; 10.14742/ajet.5761 15-26 10.14742/ajet.5761

# Reviewer report: Reviewer response for version 1

**DOI:** 10.21956/mep.21125.r35565

**Published:** 2024-01-24

**Authors:** Sherri Rudinsky

**Affiliations:** 1 Uniformed Services University, Bethesda, USA

This article describes how a simulated monitoring device (vitals display) effects students perceptions on realism and effectiveness of learning during a series of simulation scenarios. The authors accurately state in the introduction that higher fidelity, or more technology, does not always equate to a more effective learning experience and comes at a, sometimes prohibitive, cost. This pilot study provided some insight into one example where more fidelity (use of the simulated vitals monitor) appeared to positively add to the experience for a small group of students. With the positive experience being defined by student perception of realism, engagement and subjective learning. The key theme cited in the abstract - requiring less direct communication with the examiner - certainly allows for more active engagement and immersion  into the scenario and makes sense logically that this is what the students came away with. 

 A few areas where I would have liked to see more discussion regarding the methods include:

 1 - how were the facilitators trained to ensure flow of the scenario (both with and without use of the monitor). Facilitator ability to "run" the scenario contributes greatly (more than the technology itself) of the learning experience.

 2 - was there consideration to the order of the scenarios. Everyone appeared to complete scenarios without the monitors first, which may have provided a bias toward the use of monitors simply as the students felt more comfortable with simulated experiences in general by that time.

 3 - why did the authors chose different methods of data collection (survey vs interviews)? They state interviewed two (of 15) students to obtain more depth, however, they did not mention why they did not chose to interview all of them nor why those particular 2, etc. 

 The authors correctly mentioned in the limitations section that this study did not answer questions of improved performance which is in line with the overall stated learning objectives for the educational session. Ultimately, this is the "gap" in the literature - is higher fidelity more effective in achieving the educational objectives of the learning experience (improved performance outcomes), not how the students feel about the experience. Agree, this is the next required step.

Have any limitations of the research been acknowledged?

Yes

Is the work clearly and accurately presented and does it cite the current literature?

Yes

If applicable, is the statistical analysis and its interpretation appropriate?

Yes

Are all the source data underlying the results available to ensure full reproducibility?

Yes

Is the study design appropriate and is the work technically sound?

Partly

Are the conclusions drawn adequately supported by the results?

Yes

Are sufficient details of methods and analysis provided to allow replication by others?

Yes

Reviewer Expertise:

Medical Education, Simulation Education, Emergency Medicine

I confirm that I have read this submission and believe that I have an appropriate level of expertise to confirm that it is of an acceptable scientific standard, however I have significant reservations, as outlined above.

# Reviewer report: Reviewer response for version 1

**DOI:** 10.21956/mep.21125.r35151

**Published:** 2023-11-09

**Authors:** Anne D Souza

**Affiliations:** 1 Department of Anatomy, Kasturba Medical College, Manipal, Manipal Academy of Higher Education, Manipal, Karnataka, India

The manuscript addresses the comparative effect of using high-fidelity simulation with the help of a simulation observations monitor (SOM). This pilot work was carried out using a prospective study design. The manuscript is written elaborating methodology and results clearly emphasizing the key concepts. 

 There are a few areas which would require attention. Firstly, what is the rationale for using a prospective study design? The students who participated in simple simulations were made to participate again with SOM. This could lead to confounding as the same group is exposed to two interventions. A study with a two-group comparison would work well in such situations. 

 It was nice to read the student feedback on different aspects of the educational intervention. Additionally, was any test done to assess the effectiveness of the intervention? If not, what measures can be considered to evaluate learning in this situation?

 The study can be strengthened by citing more published literature on simulation-based research in undergraduate education.

Have any limitations of the research been acknowledged?

Yes

Is the work clearly and accurately presented and does it cite the current literature?

Partly

If applicable, is the statistical analysis and its interpretation appropriate?

Yes

Are all the source data underlying the results available to ensure full reproducibility?

Yes

Is the study design appropriate and is the work technically sound?

Partly

Are the conclusions drawn adequately supported by the results?

Partly

Are sufficient details of methods and analysis provided to allow replication by others?

Yes

Reviewer Expertise:

Planning and designeing learning, Medical Ethics, 3D Printing in Anatomy Education, Simulation in medical education

I confirm that I have read this submission and believe that I have an appropriate level of expertise to confirm that it is of an acceptable scientific standard, however I have significant reservations, as outlined above.
